# Soluble Klotho is associated with mortality and cardiovascular events in hemodialysis

**DOI:** 10.1186/s12882-019-1391-1

**Published:** 2019-06-11

**Authors:** Evangelos Memmos, Pantelis Sarafidis, Panagiotis Pateinakis, Apostolos Tsiantoulas, Danai Faitatzidou, Panagiotis Giamalis, Vassileios Vasilikos, Aikaterini Papagianni

**Affiliations:** 1 Department of Nephrology, Hippokration Hospital, Aristotle University of Thessaloniki, Konstantinoupoleos 49, GR54642 Thessaloniki, Greece; 2 Department of Nephrology, Papageorgiou Hospital, Thessaloniki, Greece; 3 Hemodialysis Unit, Bioclinic Thessaloniki, Thessaloniki, Greece; 4 Third Department of Cardiology, Hippokration Hospital, Aristotle University of Thessaloniki, Thessaloniki, Greece

**Keywords:** Klotho, Hemodialysis, Arteriosclerosis, Cardiovascular events, Mortality

## Abstract

**Background:**

Klotho is a transmembrane protein acting as a co-receptor for FGF-23 and thus exerts clinical actions on mineral metabolism. The association of secreted Klotho with outcomes in CKD patients is unclear. This study examined the relation between plasma Klotho and cardiovascular events in dialysis patients, accounting for common and CKD-MBD related risk factors, arterial stiffness and atherosclerotic burden.

**Methods:**

Seventy-nine chronic hemodialysis patients were observed for a median follow-up of 5.5 years. Klotho levels as well as carotid–femoral pulse wave velocity (cfPWV) and common carotid intima-media thickness (ccIMT) measurements were performed at baseline. The primary end-point was first occurrence of all-cause death, non-fatal myocardial infarction or non-fatal stroke. Secondary end-points were: (i) all-cause mortality; (ii) cardiovascular mortality; (iii) a combination of cardiovascular death, non-fatal MI, non-fatal stroke, resuscitation after cardiac arrest, coronary revascularization, heart failure hospitalization and atrial fibrillation.

**Results:**

Cumulative freedom from the primary endpoint was 31% for the low-Klotho group (≤745 pg/ml) and 53% for the high-Klotho group (logrank *p* = 0.017); HR: 2.137, 95%CI 1.124–4.065. Cumulative survival was insignificantly lower (44% vs 56%, *p* = 0.107), but cumulative cardiovascular survival (63% vs 88%, *p* = 0.029) and cumulative freedom from the cardiovascular composite outcome (18% vs 45%, *p* = 0.009) were significantly lower in the low-Klotho group. In modelled Cox-regression analysis the association of low Klotho with the primary endpoint remained significant after stepwise adjustment for cFGF3, PTH, Ca x P product, established risk factors (age, dialysis vintage, diabetes, hypertension, smoking, history of cardiovascular disease) as well as cfPWV and ccIMT [Model 6: HR:2.759, 95%CI 1.223–6.224, *p* = 0.014].

**Conclusions:**

Low Klotho is associated with cardiovascular events in hemodialysis patients, independently from factors associated with mineral-bone disease, common risk factors and intermediate outcomes, such as cfPWV and ccIMT.

## Background

Patients with chronic kidney disease (CKD) are at increased cardiovascular risk due to the presence of atherosclerosis and arteriosclerosis in the vascular wall [1, 2]. The risk of cardiovascular events increases with advancing CKD stages [3], and more than 50% of patients with end-stage renal disease on renal replacement therapy die of cardiovascular causes [4]. Current knowledge suggests that, in parallel to the “classic” risk factors of cardiovascular morbidity and mortality (i.e. age, diabetes mellitus, hypertension and smoking), patients with CKD have several other factors that may pre-dispose to cardiovascular events, such as those related to the mineral and bone disorders (CKD – MBD), anemia and the chronic inflammatory state of “uremia” [5, 6]. In addition, intermediate outcomes, such as common carotid intima media thickness (ccIMT) and pulse wave velocity (PWV), which reflect the severity of atherosclerosis and arterial stiffness respectively, have been long known as independent determinants of cardiovascular events and mortality in hemodialysis patients [5–7].

Klotho is a 130 kDa transmembrane protein, expressed predominantly in the distal convoluted tubules of the kidneys, but also in many other organs, which is involved in the regulation of human aging [8]. The extracellular domain of Klotho can undergo proteolytic cleavage by metalloproteinases and generate a 70-kDa soluble form, which is released in the circulation. Membrane Klotho binds to fibroblast growth factor receptor 1 (FGFR-1), converting it to a specific receptor for FGF-23 and thus exert its actions on mineral metabolism by regulating calcium, potassium and phosphorus excretion [9]. The biological functions of secreted Klotho may be independent of FGFR-1 and include modulation of endothelial nitric oxide synthesis, maintenance of endothelial integrity and inhibition of growth factor-1 signaling [10, 11]. Klotho knockout mice develop a syndrome similar to premature aging, with shortened life span, hyperphosphatemia, atherosclerosis and extensive vascular calcification [8]. Serum Klotho decreases from early CKD stages, partially because uremic toxins induce DNA methyltransferase protein expression, which downregulates Klotho through hypermethylation [12]. Therefore patients with CKD or on dialysis tend to generally have lower levels of Klotho than healthy individuals [13–15].

Secreted Klotho has been associated with cardiovascular events and mortality in the general population and various sub-populations (i.e. the elderly or patients with diabetes) [16, 17], however relevant studies in CKD patients are inconsistent. Two analyses from a German cohort in CKD stages 2–4 showed that Klotho is not associated with cardiovascular morbidity or all-cause mortality [18, 19]. One pilot study also shows no such associations in hemodialysis patients [20]. However, a large cohort of French hemodialysis patients, showed that patients with serum Klotho above the first quartile had a significantly reduced occurrence of cardiovascular events and death [21]. The discrepancy between the above findings in hemodialysis, can be partially explained by differences of the aforementioned cohorts concerning factors known to affect cardiovascular outcomes, including age, dialysis vintage, comorbid conditions such as hypertension, diabetes mellitus and the presence of established cardiovascular complications. Furthermore, the mechanisms through which Klotho may affect cardiovascular events are unknown. A recent study suggested that decreased Klotho levels in CKD patients were associated with increased PWV, indicating arterial stiffness as a mediatory pathway, but causality could not be established [22].

In the aforementioned studies, no evaluations of intermediate outcomes such as the degree of atherosclerosis or arteriosclerosis were performed. Thus, this study aimed to examine the possible relationship between Klotho levels and cardiovascular outcomes in hemodialysis, with simultaneous exploration of possible confounding effects from common risk factors, FGF-23 and other CKD-MBD variables, PWV and ccIMT.

## Methods

### Study design

Following a prospective cohort design, this study included patients on maintenance hemodialysis of the Dialysis Unit of the Department of Nephrology, Hippokration General Hospital and an affiliated Dialysis Unit in the city of Thessaloniki. Adult patients (i.e. > 18 years of age) undergoing maintenance hemodialysis thrice weekly for ≥3 months, were eligible to participate. All patients signed an informed consent. Exclusion criteria were: the presence of malignancy, acute infection, hepatic or chronic inflammatory disease, active antibiotic, corticosteroid or immunosuppressive treatment, previous parathyroidectomy and cardiac arrhythmias. The protocol procedures were conducted in accordance with the Declaration of Helsinki. The study was approved by the Ethics Committee of the School of Medicine, Aristotle University of Thessaloniki.

### Assessments

Baseline evaluation was performed between March and December 2010 and included a full medical history and physical examination. Demographics, dialysis-related parameters, and co-morbid conditions were recorded. Study procedures were performed in a midweek non-dialysis day, i.e. the day before the second or the third weekly session. Participants were instructed to refrain from smoking, heavy exercise, caffeine and alcohol consumption for 2 h before the examination. All measurements were performed by the same well-trained operator in a quiet room with stable air temperature (approximately 22 °C) after at least 10 min of rest, as described elsewhere [23].

Βlood pressure (BP) was measured in a sitting position in the non-fistula or the non-dominant arm after 10 min of rest with a validated oscillometric device and a cuff of appropriate size. Each patient’s systolic BP (SBP) and diastolic BP (DBP) was the mean value of three consecutive measurements within 5 min. Pulse pressure (SBP-DBP) and mean arterial pressure [SBP + 2(DBP)]/3 were estimated.

Carotid–femoral PWV was measured in each patient in a supine position with the SphygmoCor device (AtCor Medical, Sydney, Australia) which uses an applanation tonometry transducer (SPT-301, Millar Instruments, Houston, TX), as described elsewhere [23]. This instrument records carotid and femoral pulse waves with simultaneous ECG and transit time between carotid and femoral pressure waves is calculated using the foot-to-foot method. Wave ‘foots’ are identified using intersecting tangent algorithms. The distance travelled by the pulse wave is the difference between the distance from the femoral site of measurement to the sternal notch minus the distance of the carotid measurement site to the sternal notch. Pulse transit time and distance travelled by the pulse wave allowed the calculation of PWV in meters per second (m/sec) ± standard deviation. A measurement was considered valid when SD was less than 15% of PWV and the mean value of three consecutive valid measurements was used.

Ultrasonographic evaluation of intima-media thickness (IMT) was performed by an Aloka Prosound A6 device (Aloka, Tokyo, Japan), with high resolution B-mode 10 MHz transducer [24]. Bilateral common carotid IMT was visualized by longitudinal scan and far wall thickness between blood-intima and media-adventitia interfaces constituted IMT. Three measurements, 0.5, 1.0 and 2.0 cm central to the carotid bulb, were performed on each common carotid artery and thus six values were averaged to obtain the mean IMT. Arterial wall lesions protruding into the arterial lumen, with at least 0.5 mm thickness or 50% greater than the surrounding vessel wall or focal lesions with IMT > 1.5 mm, were considered as atherosclerotic plaques and were excluded from IMT evaluation.

### Laboratory analyses

Blood samples were collected on the day immediately following the study assessment day, prior to a midweek dialysis session. Routine laboratory parameters were measured directly and samples for Klotho and FGF-23 were immediately centrifuged and stored in − 80 °C. Complete blood count, calcium, phosphate, albumin, cholesterol, triglycerides, HDL-cholesterol, LDL-cholesterol, alkaline phosphatase, urea and creatinine values, were measured with routine methods in an automated laboratory analyzer (Olympus AU560, Hamburg, Germany). Serum CRP levels were measured using nephelometry, and intact PTH levels with radioimmunometric method (RIA-Immunotech, Marseille, France). Plasma Klotho was measured by a solid phase sandwich enzyme-linked immunosorbent assay (ELISA) (Immuno-Biological Laboratories Co Ltd., Fujioka-Shi, Japan). Plasma levels of intact FGF-23 and large C-terminal fragments of FGF-23 were determined using two site second-generation sandwich ELISA (human intact FGF-23 and human C-terminal FGF-23, Immutopics Inc., San Clemente, California USA. The lower detection level was 6.15 pg/mL, 1.0 pg/mL and 1.5 RU/ml for Klotho, iFGF-23 and cFGF-23 respectively. All measurements were made in duplicate and samples were measured after dilution 1:3, 1:10 and 1:20 for Klotho, iFGF-23 and cFGF-23 respectively.

### Study endpoints

Censoring was performed either on the date of the first occurrence of each studied endpoint or on 28 August 2018. The primary endpoint included all-cause death, non-fatal MI and non-fatal stroke. The secondary endpoints of the study were: (a) all-cause mortality; (b) cardiovascular mortality, which was defined as fatal MI (death by cardiovascular mechanisms occurring in 30 days and regarded as a consequence of the MI) or fatal stroke (death occurring in 30 days and regarded as a consequence or a complication of the stroke) or sudden death; (c) a combined outcome that included cardiovascular death, non-fatal MI, non-fatal stroke, resuscitation after cardiac arrest, coronary revascularization, hospitalization for heart failure or atrial fibrillation (AF).

### Statistical analysis

The study population was dichotomized based on the median plasma Klotho level (745 pg/mL). Quantitative variables are presented as mean ± standard deviation and qualitative variables as frequencies and percentages (n, %). The Shapiro-Wilk test was applied to examine the normality of distribution for continuous variables. Comparisons of continuous parameters between the groups of interest were performed with the paired Student’s t-test for independent variables or the Mann-Whitney test, as appropriate. Comparisons for categorical variables between-groups were performed with the Chi Square (χ2) test or the Fisher’s exact test. Kaplan–Meier curves and life tables were created, and the log-rank test was applied to compare the differences between the two groups of Klotho in the occurrence or freedom from the studied endpoints during follow-up. Univariate Cox regression analysis was used to evaluate the univariate association of low-Klotho with the study endpoints. Furthermore, we examined the possible confounding effect of various parameters, such as intact and c-terminal FGF23, PTH, cfPWV, ccIMT, demographic and clinical characteristics and laboratory parameters that could interfere at the association between Klotho and the primary endpoint with a modelled Cox regression analysis (enter method). Values of *p* < 0.05 (two-tailed) were considered statistically significant in all comparisons. Hazard ratios (HRs) with 95% confidence intervals (CIs) are reported. Statistical analysis was performed using the Statistical Package for Social Sciences version 25.0 (SPSS Inc., Chicago, Illinois, USA).

## Results

### Baseline characteristics and outcomes of interest

The study population consisted of 79 patients on maintenance dialysis. Baseline demographic, dialysis-related and laboratory characteristics of all participants are displayed in Table 1. The mean age of participants was 59.7 years, 63.3% were male and the mean BMI was 24.7 kg/m^2^. The primary renal disease was glomerulonephritis in 18 patients (22.8%), diabetic nephropathy in 15 patients (19.0%), obstructive nephropathy in 9 (11.4%), ADPKD in 8 (10.1%), hypertensive nephropathy in 7 (8.9%), other diseases in 3 (3.8%), while in 19 patients (24.0%) the etiology was unknown. Fifty-seven patients were on standard low-flux bicarbonate hemodialysis and 43% patients were treated with hemodiafiltration. Fifty-nine patients (74.7%) were dialyzed through a native arteriovenous fistula, 11 (13.9%) through an arteriovenous graft, while 9 patients (11.4%) had a central venous dialysis catheter. Mean Kt/V value was 1.46 ± 0.21 at baseline.

**Table 1:** Baseline demographic, anthropometric, clinical and routine laboratory characteristics of the study participants

Baseline Characteristics	Total Population, *n* = 79	Klotho≤745, *n* = 40	Klotho> 745, *n* = 39	P
Male, n(%)	50 (63.3)	26 (65.5%)	24 (61.5%)	0.750
Age (years)	59.7 ± 15.8	61.5 ± 16.1	58.1 ± 15.0	0.158
ΒΜΙ (kg/m2)	24.7 ± 4.1	25.3 ± 4.6	24.3 ± 3.6	0.307
Dialysis vintage (months)	65.5 ± 54.3	63.7 ± 52.5	70.5 ± 57.3	0.583
Hypertension, n (%)	57 (72.2%)	33 (82.5%)	24 (61.5%)	0.038
Diabetes, n (%)	17 (21.5%)	11 (27.5%)	6 (15.4%)	0.190
CVD history, n (%)	29 (36.7%)	18 (45.0%)	11 (28.2%)	0.122
Smokers, n(%)	25 (31.6%)	11 (27.5%)	14 (35.9%)	0.422
SBP (mmHg)	136.7 ± 18.4	136.2 ± 18.4	138.4 ± 17.6	0.621
DBP (mmHg)	84.1 ± 11.6	84.9 ± 12.8	84.1 ± 10.5	0.619
Pulse pressure (mmHg)	53.0 ± 13.9	49.7 ± 12.7	54.2 ± 16.2	0.322
cfPWV (m/s)	9.9 ± 2.3	10.1 ± 2.5	9.8 ± 2.1	0.565
ccIMT (mm)	0.83 ± 0.16	0.86 ± 0.16	0.79 ± 0.15	0.034
Klotho (pg/mL)	796.1 ± 236.8	661.3 ± 74.5	934.4 ± 265.7	< 0.001
iFGF23(pg/mL)	794.4 ± 1393.7	879.7 ± 1449.7	726.2 ± 1401.8	0.151
cFGF23(RU/mL)	6730.8 ± 10,113.0	6777.1 ± 11,211.3	6733.0 ± 9367.1	0.638
kt/v	1.46 ± 0.21	1,48 ± 0.20	1.44 ± 0.22	0.409
HDF	34 (43%)	15 (37.5%)	19 (48.7%)	0.314
Hemoglobin (g/dl)	11.3 ± 1.1	11.4 ± 1.3	11.3 ± 1.1	0.814
Creatinine (mg/dl)	9.24 ± 2.11	9.14 ± 1.89	9.34 ± 2.32	0.715
Urea (mg/dl)	132.7 ± 33.2	122.8 ± 29.9	138.3 ± 33.6	0.199
Albumin (g/dl)	4.02 ± 0.33	4.03 ± 0.34	3.97 ± 0.38	0.426
Cholesterol (mg/dl)	150.8 ± 38.8	151.7 ± 49.1	152.5 ± 29.3	0.433
Triglycerides (mg/dl)	140.2 ± 64.4	150.5 ± 68.6	128.9 ± 39.3	0.201
LDL-Cholesterol (mg/dl)	74.1 ± 32.1	78.5 ± 42.1	72.9 ± 23.3	0.442
HDL-Cholesterol (mg/dl)	45.1 ± 14.1	43.6 ± 13.9	48.4 ± 14.7	0.078
Serum calcium (mg/dl)	8.84 ± 0.70	8.83 ± 0.77	8.78 ± 0.60	0.316
Serum phosphate (mg/dl)	5.23 ± 1.45	5.08 ± 1.42	5.36 ± 1.56	0.834
Ca x P (mg2/dl2)	46.3 ± 13.3	46.9 ± 13.8	46.3 ± 13.1	0.869
iPTH (pmol/L)	38.5 ± 29.4	38.9 ± 31.9	39.2 ± 28.0	0.673
Alkaline phosphatase (U/L)	94.4 ± 40.2	95.9 ± 44.9	101.4 ± 40.5	0.267
CRP (mg/L)	7.34 ± 9.39	9.51 ± 13.56	5.94 ± 5.96	0.342
Intake of antihypertensive agents	57 (72.2%)	33 (82.5%)	24 (61.5%)	0.038
ESA	61 (77.2%)	30 (75%)	31 (79.5%)	0.479
Vit D and analogues	45 (57%)	22 (55%)	23 (59%)	0.721
Intravenous Iron	71 (89.9%)	35 (87.5%)	36 (92.3%)	0.479
Statins	29 (36.7%)	17 (42.5%)	12 (30.8%)	0.279

*Abbreviations*: *BMI* Body mass index, *Ca x P* Calcium x phosphorus product, *ccIMT* Common carotid Intima Media Thickness, *cFGF-23* c-terminal Fibroblast Growth Factor 23, *cfPWV* Carotid - femoral Pulse Wave Velocity, *CRP* c-reactive protein, *CVD* Cardiovascular disease, *DBP* Diastolic blood pressure, *ESA* Erythropoiesis stimulating agent, *HDL* High density lipoprotein, *HDF* Hemodiafiltration, *iFGF-23* Intact Fibroblast Growth Factor 23, *LDL* Low density lipoprotein, *iPTH* Intact parathormone, *SBP* Systolic blood pressure. Hypertension is defined as predialysis SBP ≥ 140 mmHg or DBP ≥ 90 mmHg, or use of antihypertensive drugs. Smoker status is defined as regular tobacco use or smoking cessation within the previous year. Cardiovascular disease history includes the coronary artery disease, ischemic or hemorrhagic stroke or peripheral occlusive arterial disease defined as the presence of aortic aneurysm or intermittent claudication or previous peripheral angioplasty

Study participants were divided in two groups of approximately the same size according to the median Klotho value. Forty patients (50.6%) were assigned to the low-Klotho group (levels≤745 pg/mL), and the rest in the high-Klotho group. As shown in Table 1, in univariate comparisons, there were no significant differences between the two groups for gender, age, BMI, HD vintage, kt/v, DM, all BP indices, cfPWV, cFGF-23 and iFGF-23 and most standard laboratory tests. Patients in the low-Klotho group had more commonly hypertension (*p* = 0.038), and higher values of ccIMT (0.86 ± 0.16 vs 0.79 ± 0.15, *p* = 0.034) compared with those in the high-Klotho group.

The frequencies of all study end-points are summarized in Table 2. During a median follow-up of 5.5 years, 35 (44.3%) patients died, 7 (8.9%) due to MI, 2 (2.5%) from stroke, 7 (10.1%) patients suffered a sudden death and 19 (24.1%) died from non-cardiac causes, including cancer or infection. With regards to non-fatal events, 7 (8.9%) patients suffered an MI, 5 (6.3%) a stroke, 1 (1.3%) needed resuscitation after cardiac arrest, 2 (2.5%) were hospitalised for acute decompensated heart failure, 6 (7.6%) underwent a coronary revascularization procedure and 12 (15.2%) had an episode of AF. The frequencies of study-endpoints in the two study groups are also depicted in Table 2.

**Table 2:** Absolute and relevant frequencies of outcomes of interest during follow-up in the total population and the two study groups of low- and high-Klotho

Parameter	Total Population, n = 79	Klotho≤745, n = 40	Klotho> 745, n = 39
Non fatal MI	7 (8.9%)	4 (10%)	3 (7.7%)
Fatal MI	7 (8.9%)	4 (10%)	3 (7.7%)
Non fatal Stroke	5 (6.3%)	0 (0%)	5 (12.8%)
Fatal Stroke	2 (2.5%)	1 (2.5%)	1 (2.6%)
Sudden death	7 (10.1%)	7 (17.5%)	0 (0%)
Resuscitation after cardiac arrest	1 (1.3%)	0 (0%)	1 (2.6%)
Coronary revascularization procedure	6 (7.6%)	2 (5%)	4 (10.3%)
Hospitalization for acute decompensated heart failure	2 (2.5%)	1 (2.5%)	1 (2.6%)
AF	12 (15.2%)	6 (15%)	6 (15.4%)
Cardiovascular death	16 (20.3%)	12 (30%)	4 (10.3%)
Non cardiovascular death	19 (24.1%)	9 (22.5%)	10 (25.6%)
All-cause death	35 (44.3%)	21 (52.5%)	14 (35.9%)
All-cause death or non-fatal MI or non-fatal stroke	40 (50.6%)	25 (62.5%)	15 (38.5%)
Cardiovascular death or non-fatal MI or non-fatal stroke	27 (34.2%)	18 (45%)	9 (23.1%)
Cardiovascular death, or non-fatal MI or non-fatal stroke or coronary revascularization or hospitalization for heart failure or AF	37 (46.8%)	23 (57.5%)	14 (35.9%)
All cause death, or non-fatal MI or non-fatal stroke or coronary revascularization or hospitalization for heart failure or resuscitation after cardiac arrest, or AF	48 (60.8%)	30 (75%)	18 (46.2%)

*Abbreviations*: *AF* Atrial fibrillation, *MI* Myocardial infraction

### Primary endpoint

**Fig. 1:**
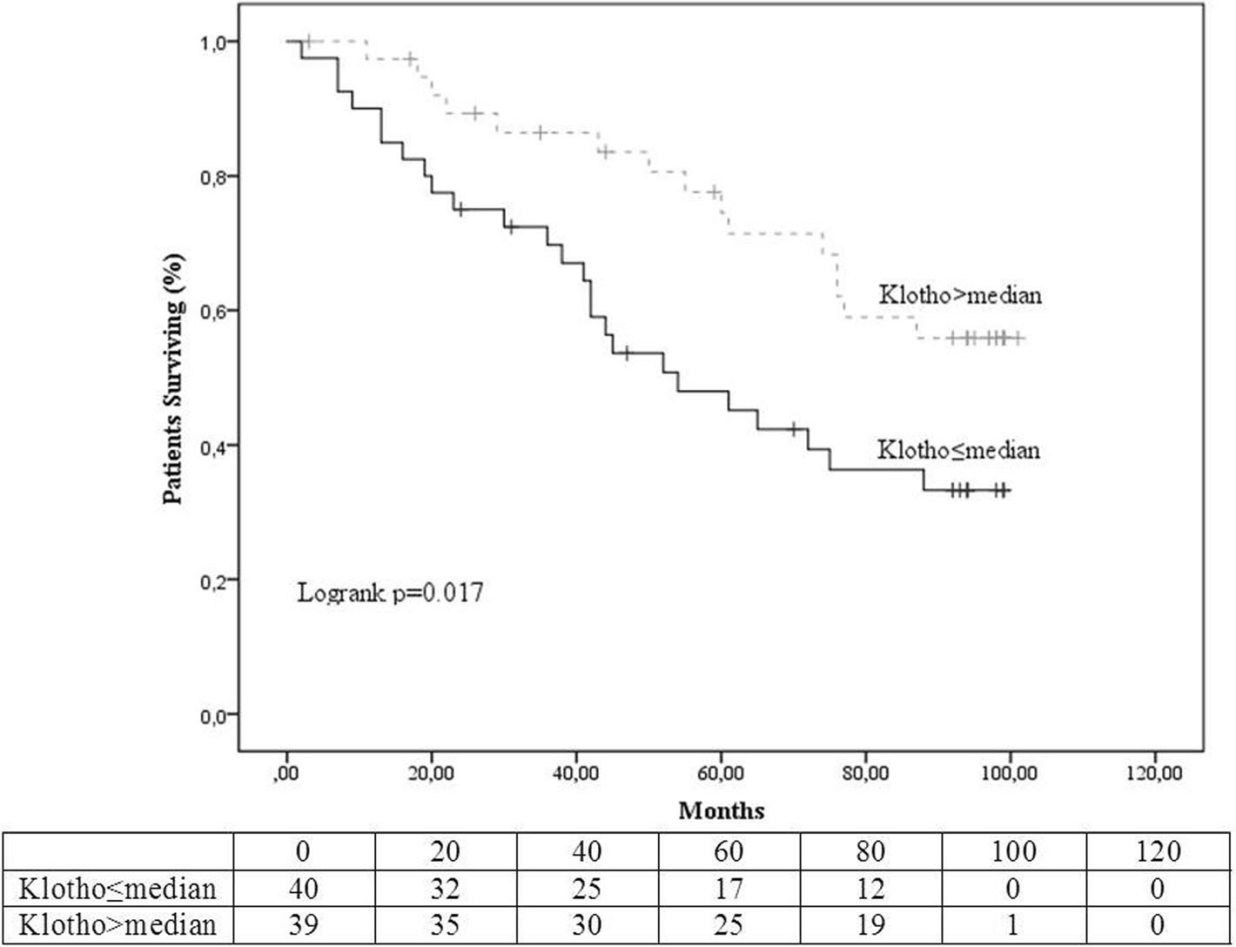
Kaplan Meier survival curves and life tables for occurrence of the primary endpoint (all-cause death or myocardial infarction or stroke)

Kaplan-Meier curves and tables of freedom from the primary endpoint (all-cause mortality, MI, stroke) between the two groups of different Klotho levels are shown in Fig. 1. The high-Klotho was the reference group in all comparisons. Cumulative freedom from the primary endpoint, during the period of observation was 31% for the low-Klotho group and 53% for the high-Klotho group, (logrank *p* = 0.017). In univariate Cox regression analysis the HR for the occurrence of the primary endpoint in the patients belonging in the low-Klotho group compared to the high-Klotho group was 2.137, 95%CI (1.124–4.065) *p* = 0.021) (Fig. 3).

### Secondary endpoints

**Fig. 2:**
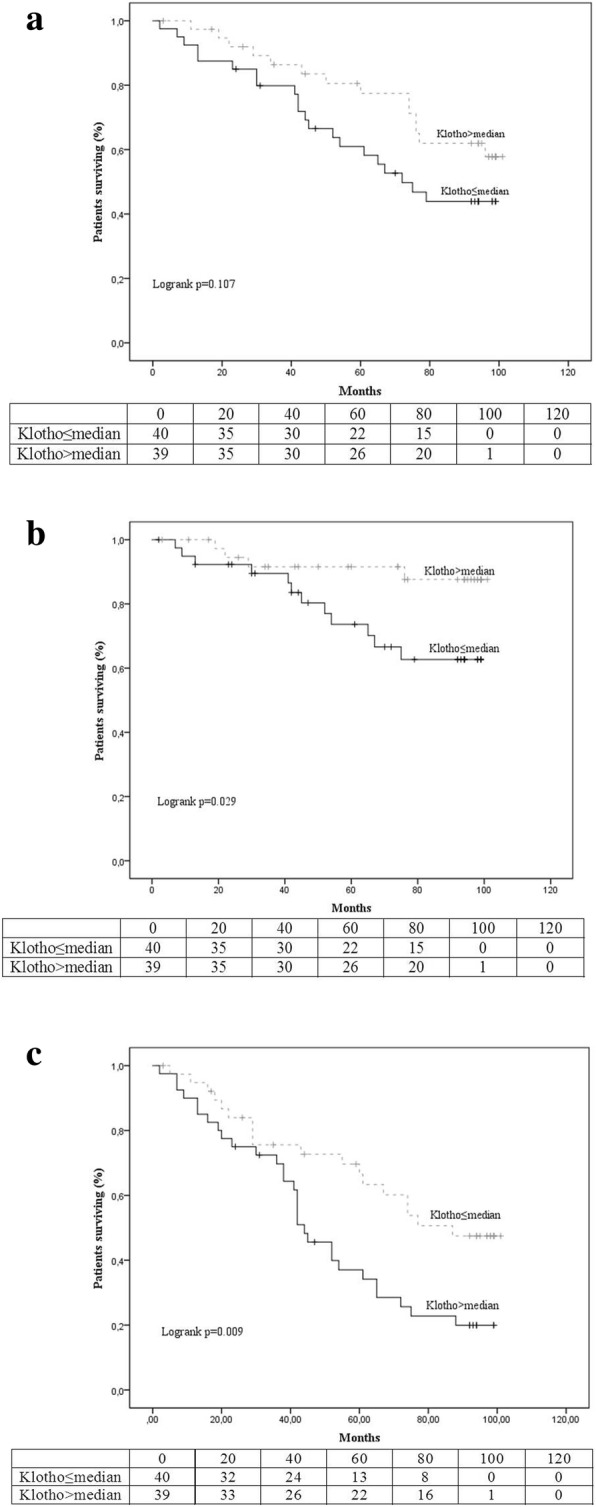
Kaplan Meier survival curves and life tables for occurrence of the secondary endpoints: **a**) all-cause mortality, **b**) cardiovascular mortality and **c**) the composite endpoint (All cause death, or non-fatal MI or non-fatal stroke or coronary revascularization or hospitalization for heart failure or resuscitation after cardiac arrest, or AF)

Figure 2, demonstrates the Kaplan-Meier curves and the tables of freedom for the three secondary outcomes. Cumulative survival was lower in the low-Klotho group but the difference between groups was not statistically significant (44% compared to 56%, logrank *p* = 0.107). Cumulative freedom from death of cardiovascular origin was 63% for patients in the low-Klotho group and 88% for those in the high-Klotho group, logrank *p* = 0.029. Similarly freedom from the composite outcome was 18 and 45%, logrank *p* = 0.009 for the low-Klotho and high-Klotho groups, respectively.

**Fig. 3:**
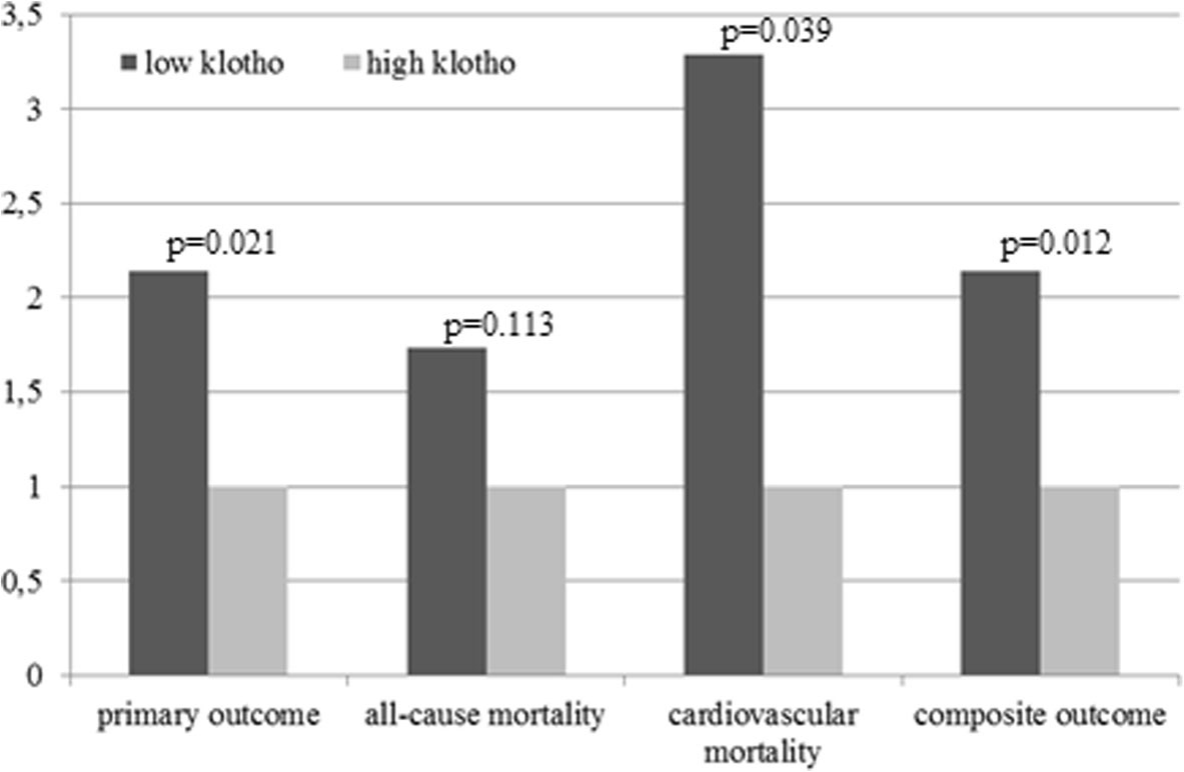
Hazard ratios for all study endpoints in the low-Klotho and the high-Klotho groups

In Fig. 3, HRs, estimated by univariate Cox regression, of the low-Klotho group compared to the high-Klotho group for all four endpoints are demonstrated, with the high-Klotho group as reference. For all-cause mortality the HR was 1.733, 95%CI 0.878–3.417, *p* = 0.113, indicating no-significant difference between groups. However, the risk of cardiovascular death (HR:3.290, 95%CI 1.059–10.217, *p* = 0.039) and the risk of the composite outcome (HR:2.139, 95%CI 1.186–3.858, *p* = 0.012), were significantly higher for patients with low compared to those with high Klotho.

### Exploration for possible confounders of the association of Klotho levels and the occurrence of death, myocardial infarction or stroke

Table 3 presents the stepwise Cox regression modelled analysis which was performed to elucidate the effect of possible confounders on the association between Klotho levels and the occurrence of the primary endpoint. As discussed above, patients with decreased Klotho levels (≤745 pg/ml) had a higher risk for the primary outcome (HR: 2.137, 95%CI 1.124–4.065, *p* = 0.021). This association remained significant after step-wise adjustment for cFGF-23, used as a dichotomous variable with the group of patients with high levels acting as the reference group (Model 2: HR: 2.081, 95%CI 1.093–3.960, *p* = 0.026). Since a strong correlation was observed between the intact and the c-terminal FGF-23 (r = 0.873, *p* < 0.001), the results are essentially the same as if iFGF-23 were used (data not shown). The association of low Klotho with the primary outcome continued to be significant as cFGF-23 and PTH (Model 3: HR:2.089, 95%CI 1.097–3.976, *p* = 0.025), and cFGF-23, PTH and Ca x P product (Model 4: HR:2.090, 95%CI 1.096–3.984, p = 0.025) were added. The association under study was not altered even after several common risk factors (age, hemodialysis vintage, diabetes mellitus, hypertension, cardiovascular disease and smoking) were included in the model (Model 5: HR:2.476, 95%CI 1.184–5.178, *p* = 0.016), and it remained significant and of the same magnitude after the addition of the two intermediate outcomes, that are established risk factors of cardiovascular disease and death, i.e. cfPWV and ccIMT (Model 6: HR:2.759, 95%CI 1.223–6.224, *p* = 0.014).

**Table 3:** Stepwise Cox regression modeled analysis for associations between low Klotho levels and the occurrence of the primary endpoint (all-cause death or myocardial infarction or stroke)

	Klotho	
	HR (95% CIs) For the low klotho group	*P* value
Model 1	2.137 (1.124–4.065)	0.021
Model 2	2.081 (1.093–3.960)	0.026
Model 3	2.089 (1.097–3.976)	0.025
Model 4	2.090 (1.096–3.984)	0.025
Model 5	2.476 (1.184–5.178)	0.016
Model 6	2.759 (1.223–6.224)	0.014

Model 1: Unadjusted; Model 2: Adjusted for cFGF-23; Model 3: Adjusted for cFGF-23 and iPTH; Model 4: Adjusted for cFGF-23, iPTH and Ca x P; Model 5: Adjusted for cFGF-23, iPTH, Ca x P, age, hemodialysis vintage and history of diabetes mellitus, cardiovascular disease (defined as history of coronary heart disease, stroke and/or peripheral vascular disease), hypertension and smoking; Model 6: Adjusted for cFGF-23, iPTH, Ca x P, age, hemodialysis vintage and history of diabetes mellitus, cardiovascular disease (defined as history of coronary heart disease, stroke and/or peripheral vascular disease), hypertension, smoking, cfPWV and ccIMT

*Abbreviations*: *Ca x P* Calcium x phosphorus product, *ccIMT* Common carotid Intima Media Thickness, *CI* Confidence intervals, *HR* Hazard ratio, *cFGF-23* c-terminal Fibroblast Growth Factor 23, *cfPWV* Carotid - femoral Pulse Wave Velocity, *iPTH* Intact parathormone

## Discussion

The present study examined the association between secreted Klotho and cardiovascular outcomes in hemodialysis patients, while simultaneously exploring the possible confounding effects caused by common and CKD-MBD related risk factors, the arterial stiffness and atherosclerotic burden. We observed that cumulative freedom from the primary endpoint, cardiovascular survival and freedom from the cardiovascular composite endpoint were significantly lower for patients in the low-Klotho group compared to those in the high-Klotho group. Overall survival was also lower in patients with low-Klotho but the between-group difference did not reach significance (*p* = 0.107), a fact that could be attributed to the low event rate. Most importantly, in stepwise Cox regression analysis the association of low Klotho with the primary outcome stayed significant after stepwise adjustment for cFGF3, PTH, Ca x P product, established risk factors (age, dialysis vintage, diabetes, hypertension, smoking, history of cardiovascular disease) as well as cfPWV and ccIMT, which are intermediate cardiovascular endpoints with known association with hard outcomes in this population.

The association of secreted Klotho with cardiovascular morbidity and mortality was originally studied in non-CKD populations. In a cohort of 804 elderly (> 65 years old) individuals observed during 6 years, patients with Klotho level in the lowest tertile (< 575 pg/mL) had an increased risk of death compared with participants in the higher quartile (> 763 pg/mL) (HR:1.78, 95% CI 1.20–2.63) [16]. In a multicenter European study [25], Klotho levels were measured in 2948 patients referred for coronary angiography irrespective of kidney function, who were then observed for almost 10 years. The study showed that Klotho does not add predictive power to cardiovascular and mortality risk assessment in patients with normal renal function, since the HRs in the fourth quartile of Klotho levels compared to the first quartile were 1.14, 95%CI 0.94–1.38, for all-cause mortality and 1.03, 95%CI 0.80–1.31, for cardiovascular mortality. In contrast, a recent study in 168 patients with type 2 diabetes, followed for 7 years, showed that low Klotho is associated with macrovascular outcomes, as patients in the highest compared to the lowest quartile of Klotho levels had HR:0.471, 95%CI 0.307–0.725, *p* = 0.001 for the combined outcome of macrovascular complications (including coronary artery disease, cerebrovascular attacks and peripheral artery occlusive disease, with significant differences present also for the individual components [17].

With regards to patients with CKD, Seiler et al. have previously, shown in 312 patients with CKD stages 2 to 4, that levels of Klotho did not predict the occurrence of death or the initiation of renal replacement therapy during 2.2 years, (HR for logarithmic transformed Klotho 1.46, 95%CI 0.14–15.95, [18]. Another study from the same group, in a cohort of 444 patients with CKD stages 2 to 4, also showed that Klotho was not associated with cardiovascular outcomes since the HR for the third versus the first Klotho tertile, was 0.75, (95%CI 0.43–1.30) [19]. In a study in 239 hemodialysis patients observed for 2.5 years, Klotho levels were not associated with mortality (HR:1.22, 95%CI 0.66–2.28 for the third compared to the first Klotho tertile), but Klotho seemed to be protective against AF [20]. In contrast, Marcais et al., showed in 769 hemodialysis patients that, patients with serum Klotho above the first quartile (≥280 ng/L) had reduced occurrence of an endpoint that combined cardiovascular events and cardiovascular death (HR:0.39, 95%CI 0.19–0.78, *p* = 0.008), compared with patients with Klotho < 280 ng/L. This effect remained significant (HR:0.86, 95%CI 0.76–0.99, *p* = 0.03) after adjustment for a number of relevant cardiovascular risk factors [21]. Two smaller studies from Asia also suggest that low Klotho is associated with higher cardiovascular risk [26, 27].

FGF-23 is a 26-kDa protein secreted mainly by osteoblasts. It can be cleaved by convertase activity and therefore intact FGF-23 and C-terminal fragments of FGF-23, which are probably inactive, are detected in plasma [28]. As FGF-23 is primarily excreted in the urine, ESRD patients on dialysis have an enormous increase in plasma levels of both the intact molecule and the C-terminal fragments [29]. In CKD patients FGF-23 secretion rises well before serum parathyroid hormone (PTH) or phosphate concentrations, because of a deficiency of the necessary Klotho cofactor. Thus, as CKD progresses, FGF-23 levels rise and Klotho levels fall [9]. FGF-23 directly increases urinary fractional excretion of phosphate, impairs the synthesis and accelerates degradation of 1,25(OH)2D and is therefore implicated in the pathogenesis of secondary hyperparathyroidism. Moreover, elevated circulating FGF-23 are independently associated with vascular dysfunction, left ventricular hypertrophy, and death [29–31].

Whether Klotho and FGF-23 act independently of each other on the cardiovascular system in CKD patients is not known. FGF-23 effects may be totally independent of Klotho, i.e. a direct action on the cardiomyocytes is described [32]. On the other hand Klotho has recently been shown to be associated with PWV, in a cross-sectional study in CKD, but causality could not be established [22]. The actions of Klotho have been extensively studied in animals, starting from the discovery of the Klotho gene in mice in 1997; Klotho-depleted animals exhibited a wide array of symptoms mimicking ageing, one of which was premature arteriosclerosis [8]. Klotho was found to have a cardioprotective effect in conditions of stress, by reducing the expression the transient receptor potential cation channels (TRPC channels) in the mouse heart [33]. Furthermore, Klotho was shown to maintain the integrity of the endothelium [11], inhibit the action of proinflammatory cytonkines such as TNF-alpha [34], and possibly have an anti-ageing effect on humans [35]. Most importantly, recent background studies have shown that Klotho protects against cardiac hypertrophy in mice independently of FGF-23 [36], whereas recombinant α-Klotho has been demonstrated to act therapeutically against uremic cardiomyopathy in mice [37].

Our study expands the aforementioned findings on the association of low-Klotho with increased cardiovascular risk, first by exhibiting that this association is independent from FGF-23 levels, or Ca x P product, both of which were associated with increased cardiovascular risk and mortality in hemodialysis [5, 31, 38]. Furthermore, in modelled analysis we observed that the association of low-Klotho with the primary outcome was additionally independent not only from classic cardiovascular risk factors but also from the intermediate endpoints of arterial stiffness, evaluated with the gold-standard method, i.e. carotid-femoral PWV, as well as the degree of atherosclerosis, evaluated by ccIMT. Of note, not only the association of low-Klotho with outcomes remained significant, but also the magnitude of the association was increasing with step-wise adjustment, going from 2.1 to 2.8-fold from univariate to the fully-adjusted model, in contrast to what commonly occurs in such analyses. Another strength of this study is the median follow-up of 5.5 years, which is the longest among the studies in hemodialysis. The main limitation is the small study sample. A detailed power estimation could not be performed as the baseline evaluation of the study took place before the publication of relevant studies in the field; to this end, this is a pilot study. This, however, did not affect our main findings, as the basic observations are very clear in terms of statistical significance, possibly due to the accumulation of events during the long follow-up. In our study, the baseline Klotho levels were on average somehow higher than in the few relevant studies in hemodialysis patients; this may due to differences in populations studied or the assays used. Lastly, we had a single evaluation of study variables (Klotho, PWV, ccIMT etc.) at baseline; as a result, they were not recorded overtime and at study-end, as commonly happens in cohort studies of this type.

## Conclusions

This pilot study showed that low Klotho is associated with increased risk of cardiovascular events and cardiovascular death in hemodialysis patients. Moreover, this association between low Klotho and the occurrence of cardiovascular outcomes was found to be independent of several important parameters, that were previously shown to be associated with increased cardiovascular risk in dialysis, including cFGF-23, and Ca x P product, age, classical cardiovascular risk factors, a history of cardiovascular disease and also, cfPWV and ccIMT. These results suggest that low plasma Klotho may accelerate (or high Klotho may protect against) cardiovascular disease in individuals with CKD through mechanisms that are distinct from known cardiovascular risk factors. Future studies are expected to shed more light in the exact role of secreted Klotho in cardiovascular outcomes in these patients.

## Abbreviations

ADPKD: Autosomal dominant polycystic kidney disease
AF: Atrial fibrillation
BMI: Body mass index
BP: Blood pressure
Ca x P: Calcium x phosphorus product
ccIMT: Common carotid intima media thickness
cFGF-23: C-terminal FGF-23
cfPWV: Carotid–femoral pulse wave velocity
CI: Confidence interval
CKD: Chronic kidney disease
CKD-MBD: Chronic kidney disease-mineral and bone disorder
CRP: C-reactive protein
DBP: Diastolic blood pressure
DM: Diabetes mellitus
ELISA: Enzyme-linked immunosorbent assay
ESRD: End-stage renal disease
FGF-23: Fibroblast growth factor 23
FGFR-1: Fibroblast growth factor receptor-1
HD: Hemodialysis
HDL: High density lipoprotein
HR: Hazard ratio
iFGF-23: Intact FGF-23
LDL: Low density lipoprotein
MI: Myocardial infarction
PP: Pulse pressure
PTH: Parathormone
SBP: Systolic blood pressure
TNF: Tumor necrosis factor
TRPC: Transient receptor potential cation channels
